# Development of a Transgenic Mouse with R124H Human TGFBI Mutation Associated with Granular Corneal Dystrophy Type 2

**DOI:** 10.1371/journal.pone.0133397

**Published:** 2015-07-21

**Authors:** Katsuya Yamazoe, Satoru Yoshida, Miyuki Yasuda, Shin Hatou, Emi Inagaki, Yoko Ogawa, Kazuo Tsubota, Shigeto Shimmura

**Affiliations:** Department of Ophthalmology, Keio University School of Medicine, Tokyo, Japan; Cedars-Sinai Medical Center; UCLA School of Medicine, UNITED STATES

## Abstract

**Purpose:**

To investigate the phenotype and predisposing factors of a granular corneal dystrophy type 2 transgenic mouse model.

**Methods:**

Human TGFBI cDNA with R124H mutation was used to make a transgenic mouse expressing human protein (TGFBI^R124H^ mouse). Reverse transcription PCR (RT-PCR) was performed to analyze TGFBI^R124H^ expression. A total of 226 mice including 23 homozygotes, 106 heterozygotes and 97 wild-type mice were examined for phenotype. Affected mice were also examined by histology, immunohistochemistry and electron microcopy.

**Results:**

RT-PCR confirmed the expression of TGFBI^R124H^ in transgenic mice. Corneal opacity defined as granular and lattice deposits was observed in 45.0% of homozygotes, 19.4% of heterozygotes. The incidence of corneal opacity was significantly higher in homozygotes than in heterozygotes (p = 0.02). Histology of affected mice was similar to histology of human disease. Lesions were Congo red and Masson Trichrome positive, and were observed as a deposit of amorphous material by electron microscopy. Subepithelial stroma was also stained with thioflavin T and LC3, a marker of autophagy activation. The incidence of corneal opacity was higher in aged mice in each group. Homozygotes were not necessarily more severe than heterozygotes, which deffers from human cases.

**Conclusions:**

We established a granular corneal dystrophy type 2 mouse model caused by R124H mutation of human TGFBI. Although the phenotype of this mouse model is not equivalent to that in humans, further studies using this model may help elucidate the pathophysiology of this disease.

## Introduction

Corneal dystrophy is a group of inherited disorder caused by progressive accumulation of deposits which affect corneal transparency and visual acuity. These deposits are not caused by inflammation, infection or trauma, but by genetic mutations. Mutations in the transforming growth factor-beta-induced (TGFBI) gene are responsible for granular, lattice and Reis-Buckler corneal dystrophy, and each dystrophy developed characteristic corneal opacities that cause visual disturbance. Among these stromal dystrophies, granular corneal dystrophy type 2 (GCD2) caused by a mutation in codon 124 of the TGFBI gene (R124H) [1] is the most common condition in Japan [2–4]. Patients with GCD2 develop corneal opacity as granular and lattice deposits that increase with age. Advanced patients suffer from severe visual impairment, especially in homozygotes [5, 6]. However, the degree and morphology of corneal opacity varies among different patients [7–9] and not all patients with the mutant gene develop corneal opacity. Previous studies have investigated predisposing factors of GCD2 including oxidative stress [10, 11], impaired autophagy [12], altered mitochondrial function [13] and increased TGFB signaling [14]. However, no definite factor that causes GCD2 is known.

Although animal models are useful in elucidating the pathogenesis and treatment of disease, no animal models of human GCD2 have been established to our knowledge. A TGFBI knockout mouse reported previously did not show a corneal phenotype [15], suggesting that mutant protein was required for the abnormal deposition characteristic of this disease. In this study, we first created a transgenic mouse model of corneal opacity caused by R124H mutation, and then investigated the characteristics and risk factors that may be associated with corneal opacity in this transgenic mouse.

## Material and Methods

### Generation of TGFBI^R124H^ mouse model

Human TGFBI cDNA with R124H mutation was used to create the transgenic mice (TGFBI^R124H^ mouse model). All animals were handled in full accordance with the ARVO Statement for the Use of Animals in Ophthalmic and Vision Research and institutional guidelines. The study was approved by the Experimental Animal Care Committee of Keio University, School of Medicine. To construct the targeting vector, a 9-kb mouse genomic DNA area including *TGFBI* gene was amplified by PCR from BAC genomic DNA clone RP23-36G24, and inserted into pBluskript II SK (+) Vector. The human *TGFBI* cDNA with R124H (CGC to CAC) mutaion was generated by PCR site-directed mutagenesis using a primer containing corresponding mutation. In detail, A part of Human *TGFBI* cDNA fragment from internal BamHI site to TAG followed by the first 17bp half of loxP sequence (a) was produced by PCR using mutation containing forward primer #1 and reverse primer #1 shown in below. The site corresponding to the R124H mutation is represented in italics with underline. The double underline in primer sequences in what follows represents restriction enzyme sites and the single underline represent anterior or posterior part of loxP sequence. The site corresponding for TAG in reverse primer #1 are represented in italics.

Forward Primer #1: 5'-AAAA$\underline{\underline{\text{GGATCC}}}$ACCACCACTCAGCTGTACACGGAC*CAC*ACGGAGAAGCTGAGGCCTGAGATGGAG -3'

Reverse Primer #1: 5'- ATGCTATACGAAGTTAT *CTA* ATGCTTCATCCTCTCTAATAAC -3'

A short double strand DNA fragment for the last 17bp half of loxP sequence followed by SmaI and SalI restriction enzyme sites (b) was produced by annealing of two oligonucleotides, forward primer #2 and reverse primer #2 shown in below. These DNA fragments (a and b) were ligated and inserted into pBluescript II SK+ (pBSIISK+) using BamHI and Sal I site (c).

Forward Primer #2: 5'- ACATTATACGAAGTTAT $\underline{\underline{\text{CCCGGG}}}$ $\underline{\underline{\text{G}}}$ -3'

Reverse Primer #2: 3'- TGTAATATGCTTCAATA $\underline{\underline{\text{GGGCCC}}}$ $\underline{\underline{\text{CAGCT}}}$ -5'

The DNA fragment for the anterior part of Human *TGFBI* cDNA from ATG to the internal Bam HI site following NotI site and Kozak sequence (d), which was produced by PCR using forward primer #3 and reverse primer #3 shown in below and cloned into pCR-BluntII-TOPO (Life Technologies Corp., carlsbad, CA) vector, was inserted into (c) using NotI and BamHI site (e). The kozak sequence and ATG in fowerd primer #3 are represented in lowercase and italics, respectively.

Forward Primer #3: 5'- AAAA $\underline{\underline{\text{GCGGCCGC}}}$ gccacc *ATG*GCGCTCTTCGTGCGGCTGCTGGCTC -3'

Reverse Primer #3: 5'- AAAA $\underline{\underline{\text{GGATCC}}}$ AACGACTCCCAGGGTCTCGTAAAGG -3'

To produce short arm (3Kbp) part followed by loxP sequence, another double stranded DNA fragment for the last 17bp of loxP sequence followed by PspOMI restriction enzyme site (f), which was produced by annealing of two oligonucleotides of forward primer #4 and reverse primer #4 shown in below, was ligated with the DNA fragment (g) produced by PCR using forward primer #5 and reverse primer #5 shown in below. The resulting DNA fragment was cloned into pBSIISK+ using SacII and PspOMI site (h).

Forward Primer #4: 5'- ACATTATACGAAGTTAT A$\underline{\underline{\text{G}}}$ -3'

Reverse Primer #4: 3'- TGTAATATGCTTCAATA T$\underline{\underline{\text{CCCGG}}}$ -5'

Forward Primer #5: 5'- AAAA $\underline{\underline{\text{CCGCGG}}}$ CCGCACGTCACACCTGGAGTGGCAAG -3'

Reverse Primer #5: 5'- ATGCTATACGAAGTTAT GGAGCTGGAGCACGCGCGGACCCAC -3'

The SacII-PspOMI fragment corresponding short arm with LoxP sequence from the construct (h) was inserted into SacII-NotI digested construct (e) and the resulting construct (i) is kozak-cDNA (R124H) flanked with loxP sequence following the short arm in pBSII SK+ vector. Subsequently, to produce construct (j), a PspOMI-NotI fragment corresponding SV40 polyA signal sequence followed by a Neo cassette flanked with FRT sequences from pBS-pA-FNF vector (UNITECH Co., Ltd., Japan) was treated for blunting and inserted into SmaI site, just behind of the rear loxP sequence, of the sonstruct (i). Next, an EcoRI-XhoI fragment from pBS-DTA vector (UNITECH Co., Ltd., Japan) corresponding DTA (diphtheria toxin fragment A) sequence was treated for blunting and inserted into behind of the long arm part (5.8Kbp, k), which was produce by PCR using forward primer #6 and reverse primer #6 shown in below and cloned into pBSIISK+ using ClaI and PspOMI site. The resulting construct (l) is the long part arm with the sequence for DTA in pBSIISK+. Finally, the SalI-digested and blunt-ended construct (j) was digested by SacII and inserted into front of the long arm of construct (l) using SacII and SmaI site.

Forward Primer #6: 5'- AAAA $\underline{\underline{\text{ATCGAT}}}$ A CAGCACGGGTAAGTCCCAGCCGCTC-3'

Reverse Primer #6: 5'- AAAA $\underline{\underline{\text{GGGCCC}}}$ ACGGGTATGTTCAGGGAACAGTGTT-3'

**Fig 1:**
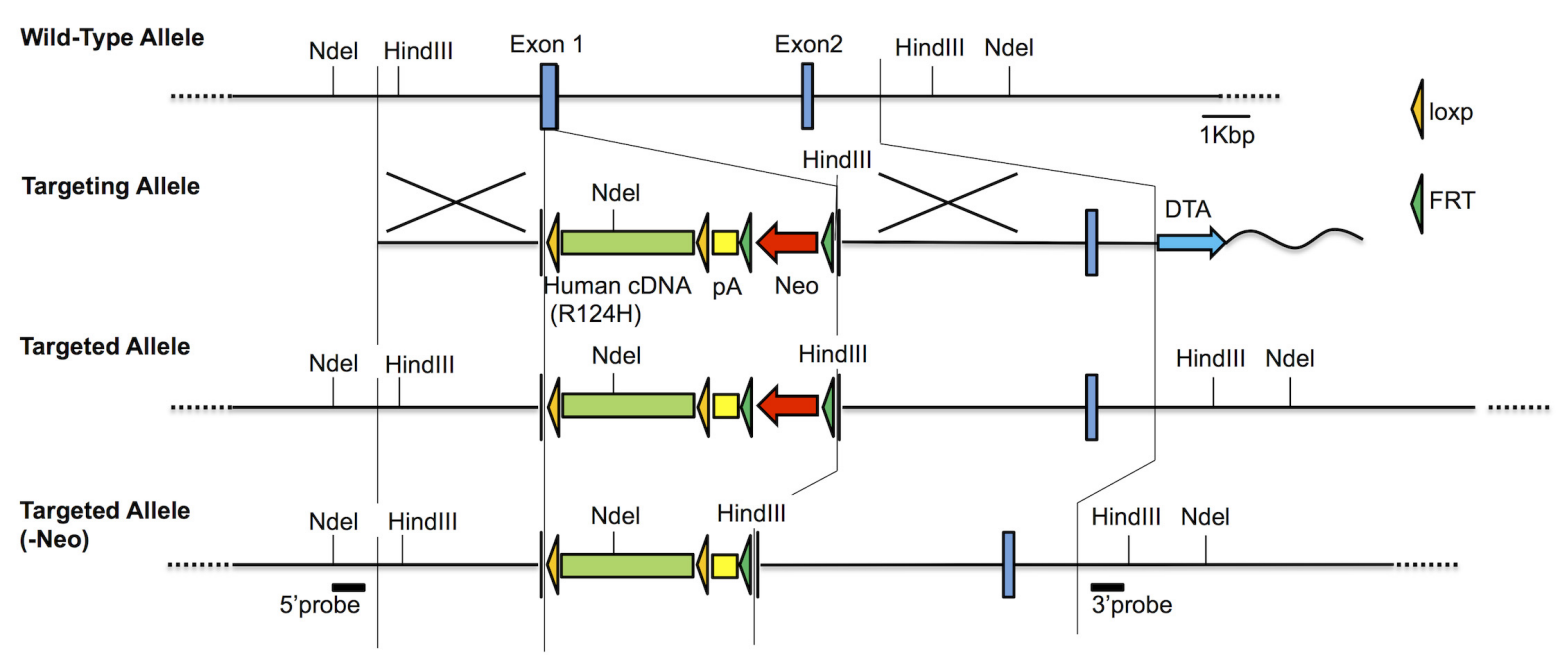
Schematic diagram for generation of the targeted TGFBI allele. Wild type allele, targeting construct, targeted allele, and Neo removed allele are shown. The human *TGFBI* cDNA sequence containing R124H mutation was knocked-in to the mouse *Tgfbi* exon 1. The detail of targeting is described in Materials and Methods.

In the resulting targeting vector (m), as illustrated in Fig 1, 130-bp of exon 1 from start codon of mouse *Tgfbi* gene was replaced by the loxP-flanked human *TGFBI* cDNA with a Kozak sequence, which contains the R124H mutation, followed by SV40 polyA signal sequence. In addition, a Neo cassette flanked with FRT was inserted after the polyA sequence for G418 selection. The full sequence of construct (e), (h), and (k) and the cDNA sequence of final construct were confirmed by sequencing. The schematic diagram of construction was shown in supplemental S1 Fig. The thermal cycling conditions for the construction were 95°C for 1 min followed by 30 cycles of 95°C for 30 sec. All PCR for these constructions were performed with PrimeSTAR HS DNA Polymerase (TAKARA BIO INC., Japan) using GeneAmp 9700 (Applied Biosystems, Foster City, CA). The thermal cycling condition for PCR from BAC genomic DNA clone was 94°C for 2 min followed by 35 cycles of 98°C for 10 sec, 58°C for 5 sec, and 72°C for 6 min. The thermal cycling condition for construction of the mutation containing cDNA was 94°C for 2 min followed by 35 cycles of 98°C for 10 sec, 58°C for 5 sec, and 72°C for 2 min.

**Fig 2:**
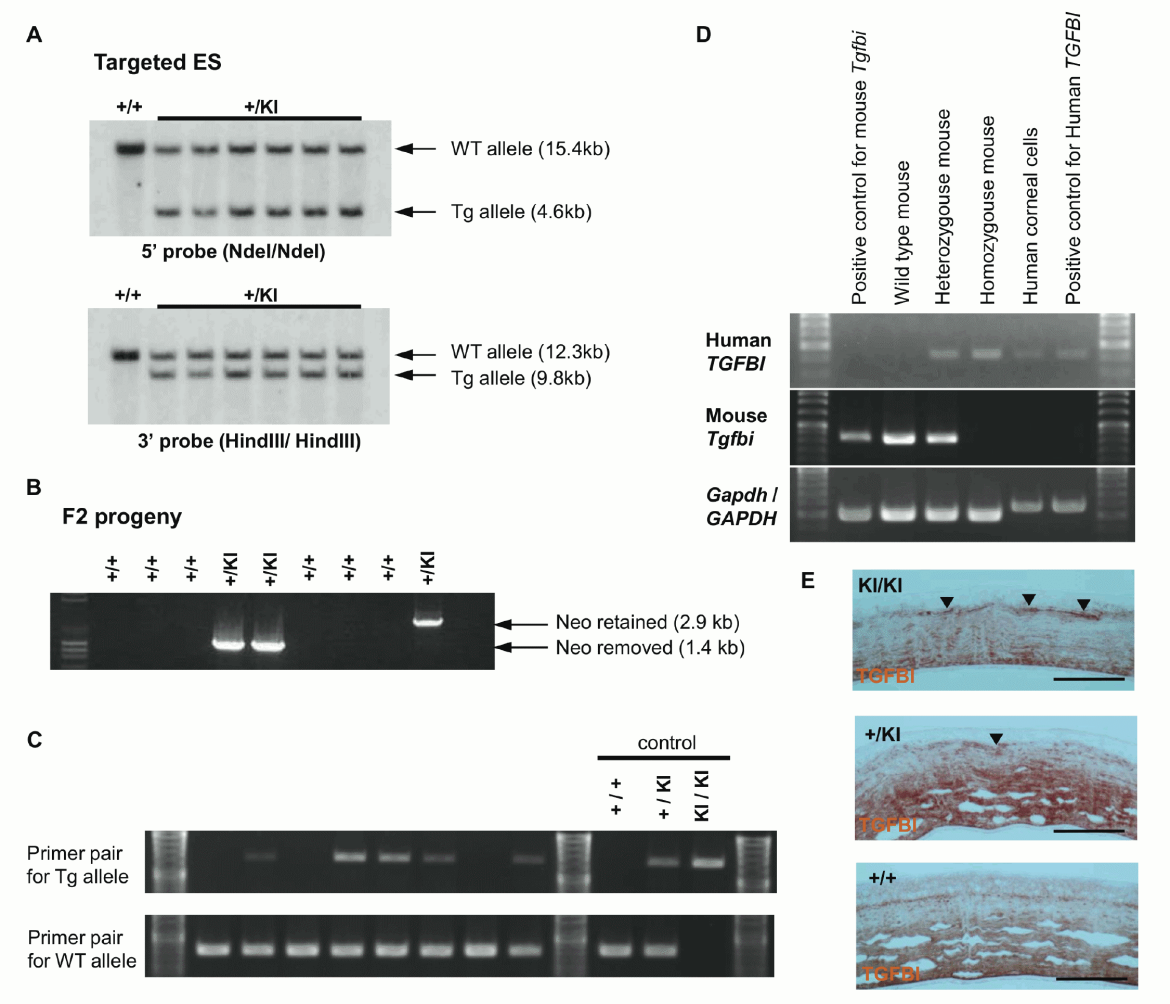
Generation of TGFBI^R124H^ Tg mice. (A) Southern blot analysis of targeted ES cells. The black bars on 5′ and 3′ outside of the targeting region in panel A indicate the locations serving as probes for genotyping by Southern blot. 15.4-kb and 4.6-kb of genomic DNA fragment is produced by Ndel digestion from wild type allele and knocked-in allele respectively, and detected by 5’ probe. 12.3-kb and 9.8-kb of genomic DNA fragment is produced by HindIII digestion from wild type allele and knocked-in allele respectively, and detected by 3’ probe. (B) Removal of Neo cassette was confirmed by genotyping PCR. The Primer pair produces 1.3-kb and 2.8-kb PCR products in absence and presence of Neo cassette, respectively. No PCR product is produced from wild type allele. (C) Representative PCR genotyping result of progenies (F12) obtained by mating *TGFBI^+/KI^* heterozygous mice with C57BL/6J mouse. Usual genotyping PCR were performed with primer pairs for knocked-in allele and wild-type allele, which give rise to 1385-bp and 787-bp of PCR products, respectively. (D) Human *TGFBI* mRNA is found in the *TGFBI^KI/KI^* homozygous mice (F12) and mouse *Tgfbi* mRNA is not. RT-PCR analysis was performed using human *TGFBI* gene and mouse *Tgfbi* gene specific primer pairs. Total RNA samples prepared from cultured mouse cells, wild-type mouse skin, human corneal cells, and cultured human cells were used as positive controls respectively. The primer pairs for human *GAPDH* gene and mouse *Gapdh* gene were used as reaction control as well. (E) TGFBI protein is found in TGFBI^KI/KI^ mouse cornea by immunohistochemistry. The sections of corneas prepared from TGFBI^KI/KI^ homozygous mouse, heterozygous mouse and wild type mouse were stained with anti-TGFBi polyclonal antibody and visualized with DAB. Dense staining is observed beneath the epithelium in KI/ KI mouse and +/KI mouse (arrow head). Scale bar = 100 um in E.

The linearized targeting vector by SacII digestion was electroporated into C57BL/6 ES cells and the targeted allele was confirmed by Southern blot (Fig 2A). The targeted ES clones were microinjected into Balb/c blastocysts and the blastocysts were transplanted into ICR foster mothers. Resulting chimeric mice were mated with C57BL/6 mice. Germ-line transmission of the targeted TGFBI allele was confirmed by PCR genotyping using genome DNA prepared from F1 offspring tail. To remove Neo cassette, the targeted F1 mice were mated with CAG-FLP mice. Finally, F3 mice without both Neo and FLP genes were obtained by mating the Neo-removed F2 mice with wild-type C57BL/6 mice. Removal of Neo (Fig 2B) and FLP sequence was also confirmed by PCR genotyping using genome DNA prepared from tail tissue. Subsequent progenies were maintained by mating with wild-type C57BL/6 mice. The genotype of these progenies was verified by PCR genotyping and a representative result was shown in Fig 2C. The sequences of primer pairs for genotyping were shown in Table 1 and the thermal cycling condition for genotyping PCR was 95°C for 1 min followed by 30 cycles of 95°C for 30 sec, 55°C for 30 sec, and 72°C for 40 sec with Blend Taq (Toyobo Co. Ltd., Osaka, Japan) using GeneAmp 9700.

**Table 1:** Oligonucleotide sequences for primer pairs used in PCR genotyping.

	Primer sequence (5’-3’)
Knocked-in (Forward)	GTGATCCACTACATTGATGAGCTAC
Knocked-in (Reverse)	GTTCAGTGACCTGTCCTATGCTC
Wild type (Forward)	CACCCTTCCCACTTTTCTACGG
Wild type (Reverse)	CTGTGTCCCCTTATCTCATCAACC
Neo (Forward)	GAACAAGATGGATTGCACGCAGGTTCTCCG
Neo (Reverse)	GTAGCCAACGCTATGTCCTGATAG
FLP (Forward)	TAGTTTGCAATTACAGTTCGAATCA
FLP (Reverse)	AGCCTTGTTGTACGATCTGACTAAG

### Cross-sectional study

Two hundred twenty-six mice including 23 homozygotes, 106 heterozygotes and 97 wild type mice were generated and examined. Left eyes of all mice were examined by stereomicroscope, and evaluated for the presence of corneal opacity. Corneal opacity was defined as (1) anterior, stromal, discrete gray-white granular deposit with or without (2) mid to posterior stromal lattice lesions, according to clinical features of human GCD2 [16]. Nineteen mice including 3 homozygotes, 8 heterozygotes and 8 wild type mice were excluded due to band keratopathy (calcium deposition), corneal edema or neovascularization, leaving 20 homozygotes, 98 heterozygotes and 89 wild type mice for this cross sectional study.

### Reverse transcription PCR

Reverse transcription polymerase chain reaction (RT-PCR) was performed to analyze TGFBI^R124H^ expression. Total RNAs from mouse corneal cells or human corneal cells were prepared using RNeasy Mini Kit (Qiagen, Germany). Human corneal cells were obtained from U.S. eye bank eyes after the central cornea was used for transplantation. First-strand cDNA samples were synthesized from the total RNAs using the Rever Tra Ace-α first-strand cDNA synthesis kit (Toyobo Co. Ltd., Osaka, Japan). Primers used for human TGFBI and mouse TGFBI are shown in Table 2. PCR was performed with Blend Taq using GeneAmp 9700. The thermal cycling conditions for RT-PCR were 95°C for 1 min followed by 30 cycles of 94°C for 30 sec, 55°C for 30 sec, and 72°C for 30 sec.

**Table 2:** Oligonucleotide sequences for primer pairs used in RT-PCR.

	Primer sequence (5’-3’)
Human Tgfbi (Forward)	CAACACGATGCTTGAAGGTAACG
Human Tgfbi (Reverse)	TCCAGAGAGATGATTGCCGAGG
Mouse Tgfbi (Forward)	CCTGCTTTCATCGTGGGTCC
Mouse Tgfbi (Reverse)	CTCCAGCACGGTATTGAGTC
Gapdh (Forward)	CAAAAGGGTCATCATCTCCGC
Gapdh (Reverse)	AGACAACCTGGTCCTCAGTGTAGC

### Histology

Formalin fixed, paraffin-embedded sections were processed using conventional histological techniques, including hematoxylin and eosion (HE) stating, Masson trichrome staining, Congo red staining, thioflavin T staining and von Kossa staining. Congo red birefrinence assay was performed. Paraffin-embedded sections were used for immunohistochemistry. Consecutive 6-um-thick paraffin-embedded sections were deparaffinized, rehydrated, and washed with phosphate-buffered saline. The sections were blocked with 10% goat serum for 30 minutes and then incubated overnight at 4°C with a rabbit polyclonal antibody raised against 208 aa length of recombinant peptides corresponding to Asn199-Gly406 of human TGFBI (Proteintech Group, Inc., Chicago, IL) and an anti-LC3 antibody (MBL, Nagoya, Japan). Immunoreactivity of the anti-TGFBI antibody was visualized with VECTASTAIN Elite ABC KIT and ImmPACT DAB Peroxidase Substrate (Vector Laboratories Inc., Burlingame, CA 94010). Immunoreactivity of the anti-LC3 antibody was visualized with secondary antibody conjugated with Cy3 (Chemicon International, Temecula, CA). After they were washed with PBS, the sections were mounted (Permafluor, Beckman Couter Inc., Miami, FL). Images were observed by a microscope (Axio Imager, Carl Zeiss Inc., Oberkochen, Germany) equipped with a digital camera (Axioncam, Carl Zeiss).

### Statistical analysis

Differences in age and sex between groups were determined using Kruskal-Wallis test or chi-square test. Differences in the incidence of typical corneal opacities between groups were determined using Fisher’s exact test. Risk factors associated with incidence of corneal opacity were determined using a logistic regression. A P-value of <0.05 was considered statistically significant. Multivariate logistic regression analyses were performed for all variables. All statistical analyses were performed with SSRI software (SSRI, Tokyo, Japan).

## Results

### Phenotype of TGFBI^R124H^ mice

RT-PCR and immunohistochemistry confirmed human TGFBI expression in the TGFBI^R124H^ mouse (Fig 2D). Heterogyzous mice expressed both mouse *Tgfbi* and human *TGFBI* genes. The sections of corneas prepared from TGFBI^KI/KI^ homozygous mouse, heterozygous mouse and wild type mouse were stained with anti-TGFBi polyclonal antibody and visualized with DAB (Fig 2E). Subepithelial stroma was relatively strongly stained in homozygotes and heterozygotes.

As described in materials and methods, the antibody we used is a rabbit polyclonal antibody raised against human TGFBI and human TGFBI are 91% identical with mouse TGFBI in amino acid sequence. Therefore, this antibody reacts with mouse TGFBI as well. Some monoclonal antibodies for human TGFBI are commercially available but they also react with mouse TGFBI protein.

No antibodies are available which can distinguish human and mouse TGFBI as far as we know. However, in KI/KI homozygous mouse, endogenous TGFBI is not expressed because the human TGFBI was knocked-in to replace the endogenous gene as shown by RT-PCR (Fig 2D). For these reasons, the immunostaining signal in KI/KI homozygous mouse is due to the human *TGFBI* transgene although staining results were similar to heterozygous mouse (Fig 2E).

**Fig 3:**
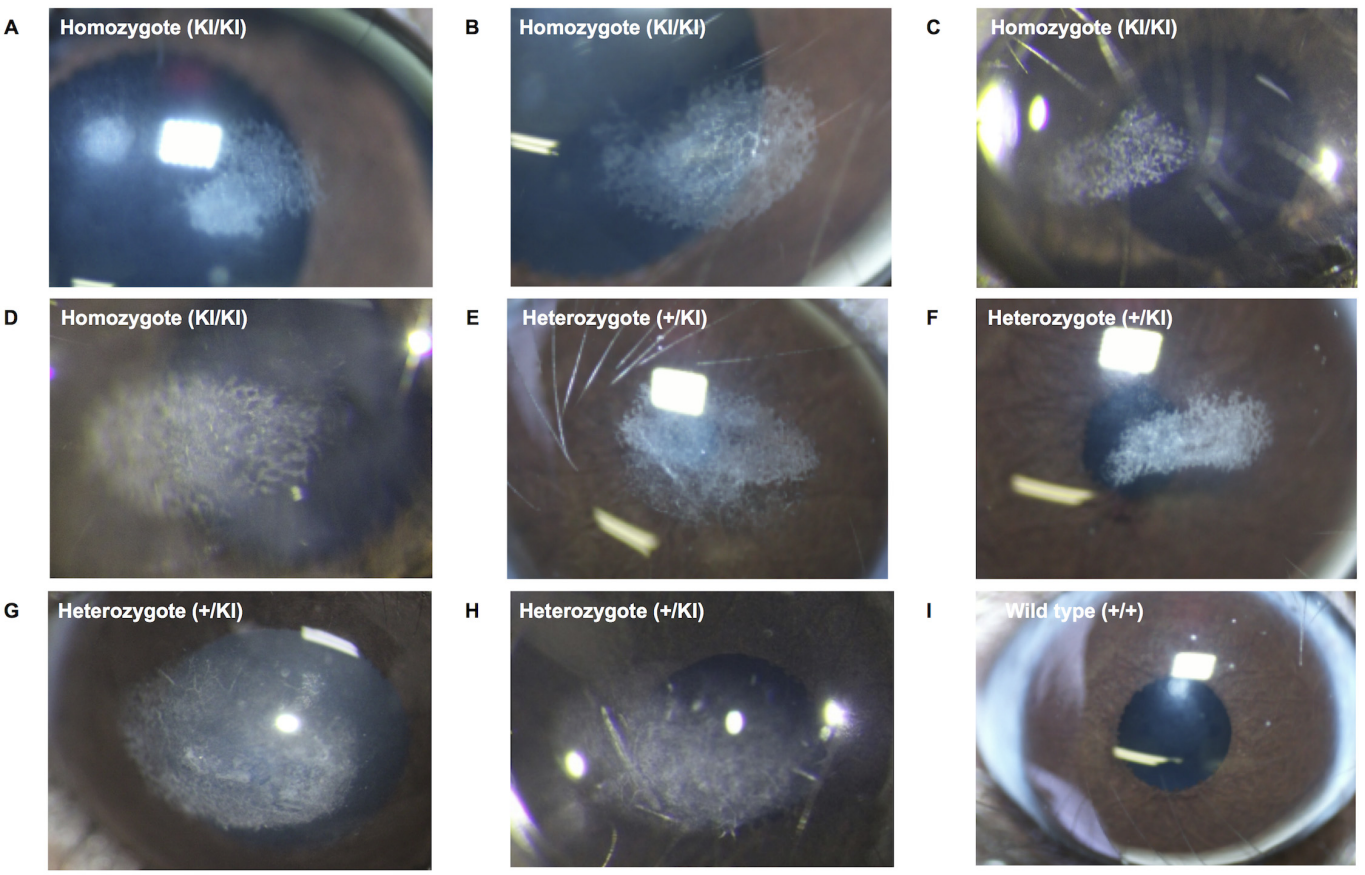
Phenotype of TGFBI^R124H^ mice. Granular and lattice deposits without corneal edema and neovascularization were observed in the center of the cornea in homozygous (52 weeks, female, A, B, 78 weeks, male, C, D) and heterozygous (52 weeks, female, E, F, 94 weeks female, G, H) mice. Wild type mice had clear corneas (I).

**Fig 4:**
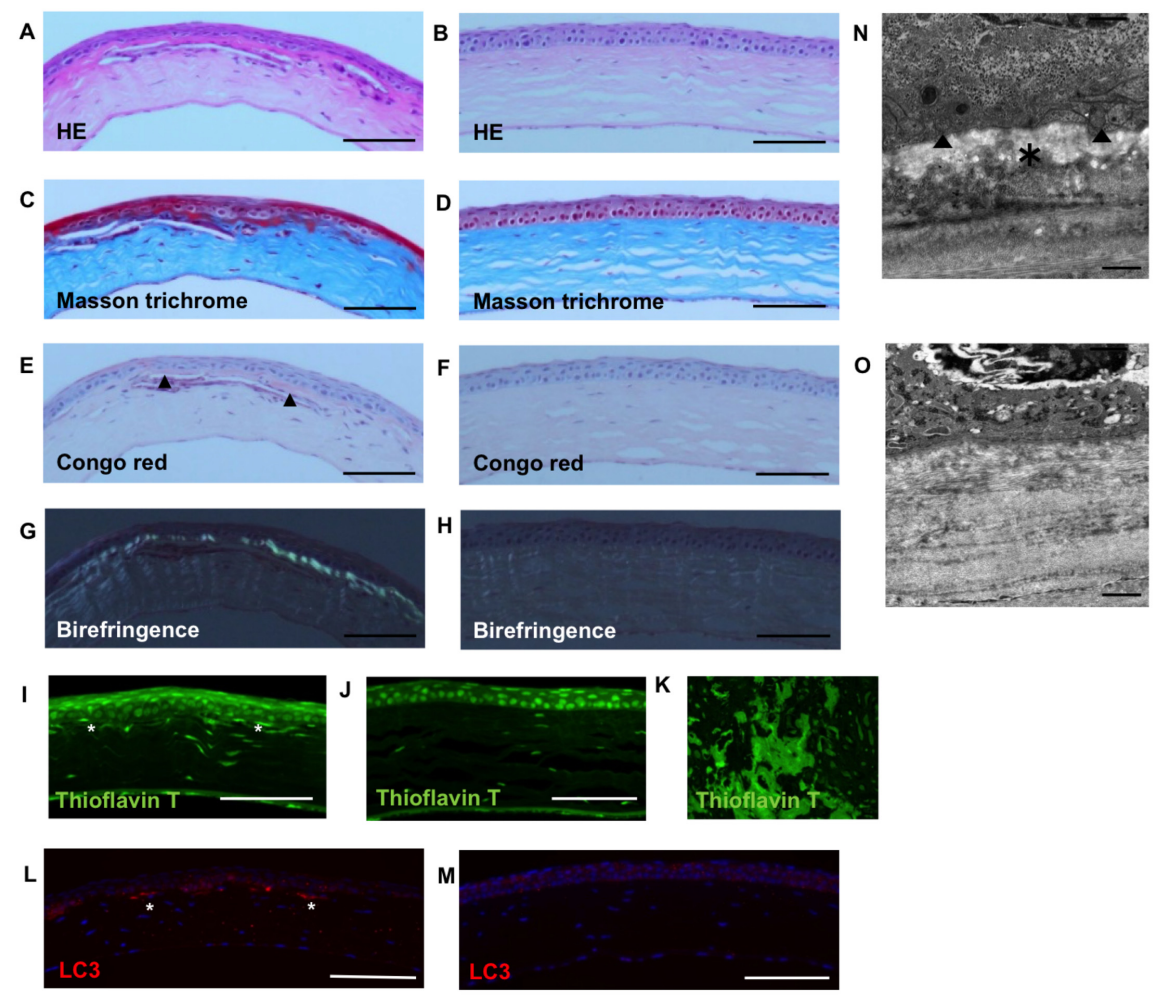
Histology of TGFBI^R124H^ mice. In homozygotes, HE staining did not show signs of inflammation in the cornea (A) as well as wild type mice (B), while Masson trichrome staining showed red deposits in the anterior cornea (C) in contrast to wild type mice (D). Congo red stating showed red deposits (arrow head) in the anterior cornea (E) and birefringence of the area was observed (G) in contrast to wild type mice (F, H). Subepithelial stroma was also stained with thioflavin T (*) in homozygotes (I) in contrast to wild type mice (J). Positive control: Human renal amyloidosis (K). Non-specific staining of cell nuclei is observed in both samples and positive control. Immunohistochemical examination with LC3 showed subepitheilaicl stromal staining (*) in homozygotes (L) in contrast to wild type mice (M). Examination with electronic microscopy revealed subepithelial deposits (*) in heterozygotes (N) in contrast to wild type mice (O). Autophagosomes (arrow head) were observed around the deposits. Scale bar = 100 um in A—J, 1 um in K, L.

Stereomicroscope examination of homozygotes and heterozygotes revealed granular deposits with or without lattice deposits in the center of the cornea (Fig 3A–3H). There were no wild type mice with typical corneal opacity (Fig 3I). In homozygotes, HE staining did not show signs of inflammation in the cornea (Fig 4A). Masson trichrome staining (Fig 4C) and Congo red stating (Fig 4E) were positive, and birefringence of the area was also observed (Fig 4G). Subepithelial stroma was also stained with thioflavin T in homozygotes (Fig 4I) in contrast to wild type mice (Fig 4J). Immunohistochemical examination with LC3, a marker of autophagy activation revealed that subepithelial stroma was stained in homozygotes (Fig 4L). Electronic microscopy of heterozygotes revealed subepithelial deposition of amporphous material (Fig 4N), but not in wild type corneas (Fig 4O). Autophagosomes (arrow head) were observed around the deposits.

### Demographic characteristics

Mouse demographics are shown in Table 3. There were no statistical differences in age and sex between 3 groups. Total incidence rates of corneal opacity were 45.0% in homozygotes, 19.4% in heterozygotes, and 0.0% in wild type mice. These differences were statistically significant. Bilateral opacity was observed in 8 homozygotes (88.9%) and 9 heterozygotes (47.4%). Age-associated incidence is shown in Table 4. A higher incidence of corneal opacity was observed in aged mice in each group.

**Table 3:** Demographic features.

	Homozygotes	Heterozygotes	Wild type
N	20	98	89
Age (weeks)	61.7 ± 33.3	55.7 ± 21.8	49.3 ± 18.2
Sex (male/female)	14/6	54/44	51/38

**Table 4:** Incidence of corneal opacity.

	Homozygotes		Heterozygotes		Wild type	
Age	N	(%)	N	(%)	N	(%)
<40 weeks	1/6	16.7	2/25	8.0	0/35	0.0
40–59 weeks	3/7	42.9	5/40	12.5	0/39	0.0
60–79 weeks	2/3	66.6	4/14	28.6	0/8	0.0
≥80 weeks	3/4	75.0	8/19	42.1	0/7	0.0

### Factors affecting incidence of corneal opacity

Univariate logistic regression analyses in homozygous and heterozygous mice between the occurrence of corneal opacity and factors including genotype and sex revealed that genotype was associated with the incidence of corneal opacity. Homozygotes were more prone to develop corneal opacity than heterozygotes (OR, 3.40, 95%CI. 1.23–9.37, p = 0.0179). Sex was not associated with the incidence of corneal opacity (OR, 0.61, 95%CI. 0.26–1.42, p = 0.2484). Multivariate logistic regression analyses also revealed that genotype associated with the incidence of corneal opacity (OR, 3.97, 95%CI. 1.38–11.39, p = 0.0105). Sex was not associated with the incidence of corneal opacity (OR, 0.50, 95%CI. 0.20–1.23, p = 0.1287).

## Discussion

We established a GCD2 mouse model caused by R124H mutation of TGFBI. Granular opacities were observed in the center of cornea and lattice-shaped opacities were also observed in some eyes. There were no mice with only lattice-type lesions. These opacities were not accompanied by corneal edema or neovasucularization. The incidence of granular opacities was significantly higher in homozygotes than in heterozygotes. Multivariate logistic regression analyses also revealed that genotype was associated with the incidence of corneal opacity. Additionally, a higher incidence of corneal opacity was observed in aged mice in each group as in human cases [17]. Masson-trichrome and Congo red staining showed deposits of hyaline and amyloid in the anterior cornea. These results suggest corneal opacity of this mouse model reflects the findings of GCD2 in humans.

However, this mouse model may differ from human cases in the severity of corneal opacity. In human cases, the opacity is usually severe and often causes visual impairment in homozygotes [5, 6]. In our mouse model, homozygotes were not necessarily more severe than heterozygotes. We do not know the reason for this, but the small diameter of mouse corneas may be one possible explanation. In addition, the detection of amyloid was difficult in our mouse model. Amyloid deposition may not be a major finding in this model, suggesting a slightly different phenotype to human disease. This may be a limitation of the model, however, our TGFBI^R124H^ mouse is the first to show any TGFBI-related phenotype to our knowledge.

R124H mutation shows incomplete penetrance, and patients with this mutation do not necessarily develop corneal opacity typical to GCD2. Cao *et al* reported that 4 out of 11 individuals with the mutation had phenotypic features consistent with diagnosis of GCD2 [18]. In this mouse model, not all mutant mice developed corneal opacity as in cases with human GCD2. Therefore, different epi-genetic and environmental factors may influence the phenotype in both humans and our mutant mouse. While typical lesions were not observed in wild type mice, non-specific opacification due to calcium deposition (band keratopathy) was observed in approximately 5% of mice (S2 Fig). Diagnosis of homozygotes and heterozygotes may be difficult macroscopically when band keratopathy is present.

Recently, several factors were reported to be associated with occurrence of GCD2. Accelerated deposits after LASIK in GCD2 were reported in several studies [19–21]. This suggests that traumatic stress of the cornea may affect corneal deposition. We performed epithelial ablation using a Algerbrush in heterozygotes and wild type mice without corneal opacity. However, this intervention did not cause corneal granular opacity (data not shown). Although we were not able to induce enhanced disease in a wound healing experiment performed under our experimental conditions, excimer photoablation in this model may be an interesting study.

Other studies showed that corneal deposits in GCD2 include the TGFBI protein [22], and that the R124H mutation alone did not affect the stability of TGFBI protein [23]. Impaired degradation of mutant TGFBI may therefore cause deposition of the abnormal TGFBI protein [22, 24]. The mutant TGFBI protein was also shown to be degraded differently in a limited proteolysis experiment *in vitro* [25]. Similarly, protease inhibitors may accelerate disease, in which case enhanced protease activity may be a therapeutic option. However, interventional studies designed to test this hypothesis using aprotinin and tissue inhibitor of metalloproteinase (TIMP) injected into corneal stroma did not yield positive results (data not shown). Impaired autophagy and delayed autophagic clearance of TGFBI protein in *in vitro* experiments were also reported and activation of autophagy was suggested to be a therapeutic strategy [12]. In our mouse model, immunohistochemical study revealed that subepithelial stroma of homozygote was stained with anti-LC3 antibody, and this result support our disease model. Benzalkonium chloride accelerates the formation of the amyloid of corneal dystrophy-associated peptides *in vitro* [26]. We administered benzalkonium chloride 0.001% eye drops two times daily for two weeks. However, acceleration of disease both in age of onset or extent of opacification was not observed (data not shown). While these may be a problem of experimental design, it is more probable that no single factor is involved. There is also a possibility that the type of protein aggregation in this mouse model may not be identical to human GCD2. Further studies using this mouse model may help elucidate the pathophysiology of the disease.

In summary, we established a human granular corneal dystrophy type 2 mouse model caused by R124H mutation of TGFBI. While none of our interventional studies enhanced the progression of disease, the cross-sectional study revealed that genotype and age were associated with the incidence of corneal opacity. Further studies may reveal multiple risk factors that may lead to the development of preventive therapy for this disease.

## Supporting Information

S1 Fig Schematic diagram of the targeting vector construction. A part of Human *TGFBI* cDNA fragment from internal BamHI site to TAG followed by the first 17bp half of loxP sequence (a) was produced by PCR using a mutation containing primer pair. The site corresponding to the R124H mutation (CAC) is represented with asterisk. The DNA fragment (a) and a short double strand DNA fragment for the last 17bp half of loxP sequence followed by SmaI and SalI restriction enzyme sites (b) were ligated and inserted into pBluescript II SK+ (pBSIISK+) using BamHI and Sal I site (c). The DNA fragment for the anterior part of Human *TGFBI* cDNA from ATG to the internal Bam HI site following NotI site and Kozak sequence (d) produced by PCR was inserted into (c) using NotI and BamHI site (e). To produce short arm (3Kbp) part followed by loxP sequence, another double stranded DNA fragment for the last 17bp of loxP sequence followed by PspOMI restriction enzyme site (f) was ligated with the DNA fragment (g) produced by PCR. The resulting DNA fragment was cloned into pBSIISK+ using SacII and PspOMI site (h). The SacII-PspOMI fragment corresponding short arm with LoxP sequence from the construct (h) was inserted into SacII-NotI digested construct (e) and the resulting construct (i) is kozak-cDNA (R124H) flanked with loxP sequence following the short arm in pBSII SK+ vector. Subsequently, to produce construct (j), a PspOMI-NotI fragment corresponding SV40 polyA signal sequence followed by a Neo cassette flanked with FRT sequences was inserted into SmaI site, just behind of the rear loxP sequence, of the sonstruct (i). Next, an EcoRI-XhoI fragment corresponding DTA (diphtheria toxin fragment A) sequence was inserted into behind of the long arm part (5.8Kbp, k) produced by PCR and cloned into pBSIISK+. The resulting construct (l) is the long part arm with the sequence for DTA in pBSIISK+. Finally, SalI-SacII fragment from construct (j) was inserted into front of the long arm of construct (l) using SacII and SmaI site. The resulting construct (m) was used as the targeting vector and linearization was performed by SacII digestion. The full sequence of construct (e), (h), and (k) and the cDNA sequence of final construct were confirmed by sequencing. Detailed oligonucleotides sequences were described in Materials and Methods.
(TIFF)

S2 Fig Histology of wild type mouse with band keratopathy. Non-specific opacification due to calcium deposition (band keratopathy) was observed in approximately 5% of mice (A). HE staining did not show signs of inflammation in the cornea (B). Masson trichrome staining did not showed red deposits in the anterior cornea (C). Congo red stating did not showed red deposits in the anterior cornea (D) and birefringence of the area was not observed (E). Von Kossa staining showed black deposits in the anterior cornea (F). Scale bar = 100 um in B—F.
(TIFF)
