# Supplementary figures and images for: Development of a Transgenic Mouse with R124H Human TGFBI Mutation Associated with Granular Corneal Dystrophy Type 2
Source: PLoS One. 2015 Jul 21;10(7):e0133397. doi: 10.1371/journal.pone.0133397 (PMC4511001; doi:10.1371/journal.pone.0133397)

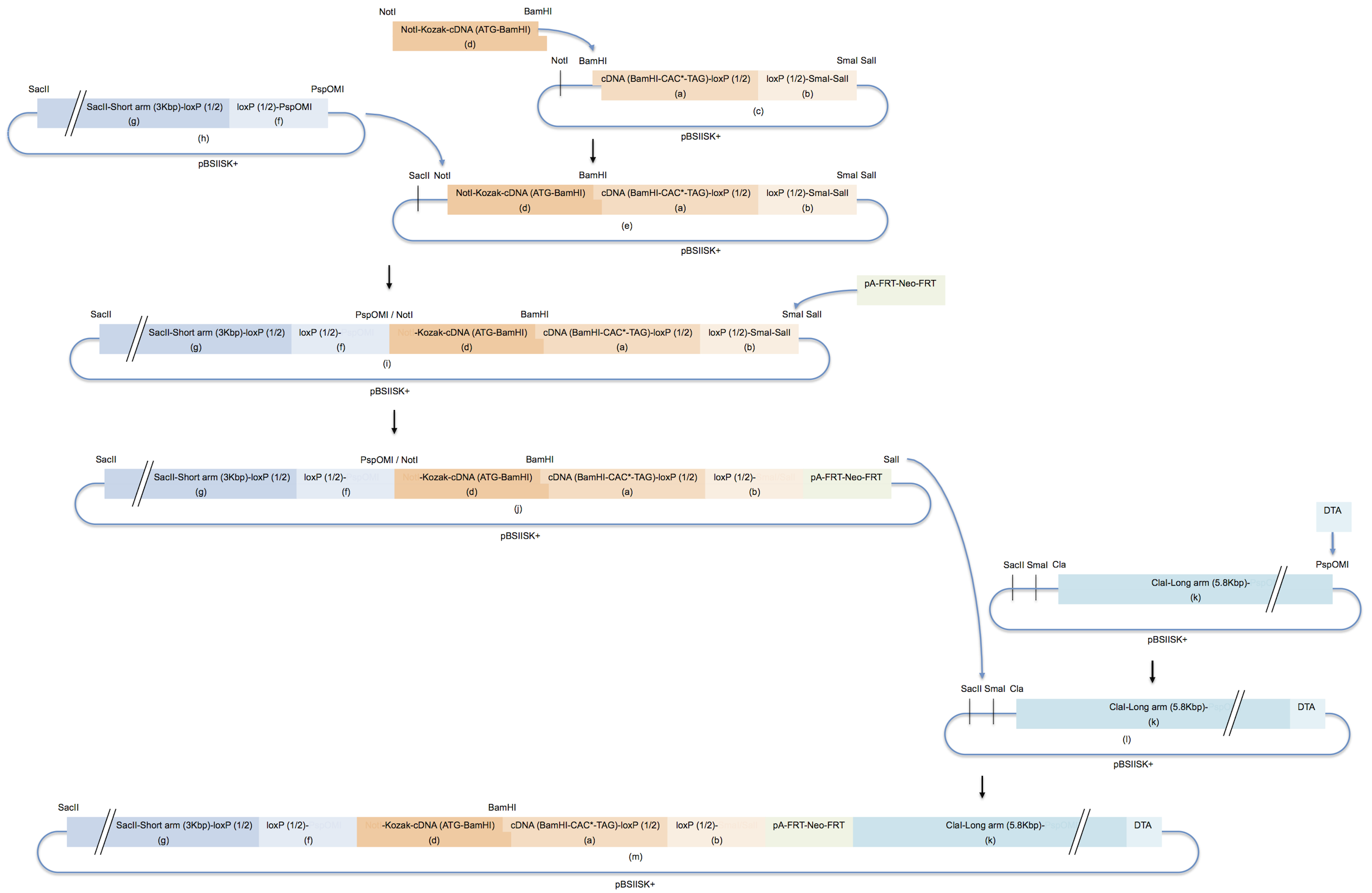

Supplement: S1 Fig — A part of Human TGFBI cDNA fragment from internal BamHI site to TAG followed by the first 17bp half of loxP sequence (a) was produced by PCR using a mutation containing primer pair. The site corresponding to the R124H mutation (CAC) is represented with asterisk. The DNA fragment (a) and a short double strand DNA fragment for the last 17bp half of loxP sequence followed by SmaI and SalI restriction enzyme sites (b) were ligated and inserted into pBluescript II SK+ (pBSIISK+) using BamHI and Sal I site (c). The DNA fragment for the anterior part of Human TGFBI cDNA from ATG to the internal Bam HI site following NotI site and Kozak sequence (d) produced by PCR was inserted into (c) using NotI and BamHI site (e). To produce short arm (3Kbp) part followed by loxP sequence, another double stranded DNA fragment for the last 17bp of loxP sequence followed by PspOMI restriction enzyme site (f) was ligated with the DNA fragment (g) produced by PCR. The resulting DNA fragment was cloned into pBSIISK+ using SacII and PspOMI site (h). The SacII-PspOMI fragment corresponding short arm with LoxP sequence from the construct (h) was inserted into SacII-NotI digested construct (e) and the resulting construct (i) is kozak-cDNA (R124H) flanked with loxP sequence following the short arm in pBSII SK+ vector. Subsequently, to produce construct (j), a PspOMI-NotI fragment corresponding SV40 polyA signal sequence followed by a Neo cassette flanked with FRT sequences was inserted into SmaI site, just behind of the rear loxP sequence, of the sonstruct (i). Next, an EcoRI-XhoI fragment corresponding DTA (diphtheria toxin fragment A) sequence was inserted into behind of the long arm part (5.8Kbp, k) produced by PCR and cloned into pBSIISK+. The resulting construct (l) is the long part arm with the sequence for DTA in pBSIISK+. Finally, SalI-SacII fragment from construct (j) was inserted into front of the long arm of construct (l) using SacII and SmaI site. The resulting construct (m) was used a [file pone.0133397.s001.tiff]

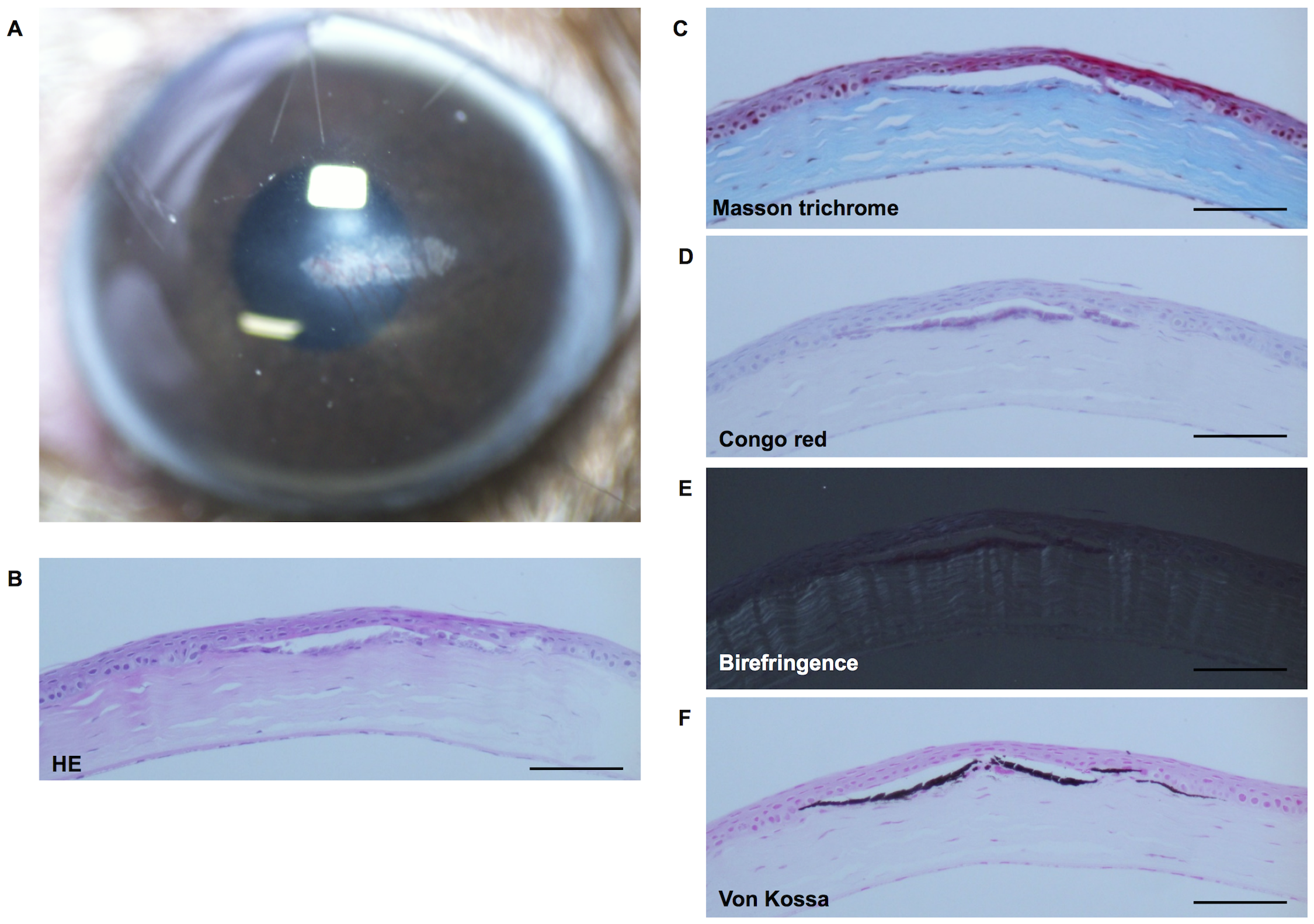

Supplement: S2 Fig — Non-specific opacification due to calcium deposition (band keratopathy) was observed in approximately 5% of mice (A). HE staining did not show signs of inflammation in the cornea (B). Masson trichrome staining did not showed red deposits in the anterior cornea (C). Congo red stating did not showed red deposits in the anterior cornea (D) and birefringence of the area was not observed (E). Von Kossa staining showed black deposits in the anterior cornea (F). Scale bar = 100 um in B—F. (TIFF) [file pone.0133397.s002.tiff]
